# Ultrastructure of the fertilized egg envelope from *Melanotaenia praecox*, Melanotaeniidae, Teleostei

**DOI:** 10.1186/s42649-021-00052-z

**Published:** 2021-04-01

**Authors:** Joon Hyung Sohn, Dong Heui Kim

**Affiliations:** 1 Institute of Lifestyle Medicine, Yonsei University Wonju College of Medicine, Wonju, 26426 South Korea; 2 Department of Environmental Medical Biology, Yonsei University Wonju College of Medicine, Wonju, 26426 South Korea

**Keywords:** Egg envelope, Fertilized egg, *Melanotaenia praecox*, Melanotaeniidae, Ultrastructure

## Abstract

We examined the morphology of fertilized egg and ultrastructures of fertilized egg envelopes of dwarf rainbowfish (*Melanotaenia praecox*) belong to Melanotaeniidae using light and electron microscopes. The fertilized eggs were spherical with adhesive filament, transparent, demersal, and had a narrow perivitelline space and small oil droplets. The size of fertilized egg was 1.02 ± 0.18 mm (*n* = 30), and there were two kinds of adhesive filament on the fertilized eggs. The long and thick (diameter 12.22 ± 0.52 μm, *n* = 20) adhesive filaments were only at the area of animal pole, and short and thin (diameter 1.99 ± 0.23 μm, *n* = 20) adhesive filaments were around the long filaments. A micropyle was conical shaped with adhesive filament and located near the animal pole of egg. The outer surface of fertilized egg was rough side. Also, the total thickness of the fertilized egg envelope was about 7.46 ± 0.41 μm (*n* = 20), the fertilized egg envelope consisted of two layers, an inner lamellae layer and an outer layer with high electron-density. And the inner layer was 8 layers. Collectively, these morphological characteristics and adhesive property of fertilized egg with adhesive filaments, and ultrastructures of micropyle, outer surface, and section of fertilized egg envelope are showed species specificity.

## Introduction

Dwarf rainbowfish (*Melanotaenia praecox* Weber & Beaufort, 1922) is a teleost belong to family Melanotaeniidae, order Atheriniformes and class Actinopterygii, and habits Mamberamo River in northern Irian Jaya, Indonesia (Fishbase contributors 2021). This species is an omnivorous species, the males tend to be brighter in color and have deeper bodies than the females (Tappin 2016). Hatching begins 119.50 h post-fertilization at 28 °C and newly hatched larvae were excellent swimming activity (Radael et al. 2014).

The eggs of teleost are surrounded by an egg envelope, it has some functions including protections of physical, chemicals and biological factors such as bacteria, fungus and virus, and selective or passive transports (Laale 1980; Grierson and Neville 1981; Harvey et al. 1983; Cameron and Hunter 1984). In teleost, there are two types of fertilized eggs, adhesive and non-adhesive types. The adhesive type is one among these three types, with adhesive filament or protuberance, or fertilized egg is adhesive in itself without adhesive structures (Kim et al. 1996; Kwon et al. 2017, Choi et al. 2019).

The structure of fertilized egg has been showed family specificity because the fertilized eggs had same morphology in same family under the stereo microscopic observation. But ultrastuctures of outer surface, micropyle, special adhesive structures and section of fertilized egg envelope were showed species, genus or family specificities (Kim et al. 1996, 1998; Kwon et al. 2015, 2017, Choi et al. 2019). This ultrastructural difference of fertilized egg envelope has been known to be related with physical and chemical properties of water environment, location of habitat (Ivankov and Kurdyayeva 1973; Stehr and Hawkes 1979; Laale 1980) and species variation (Joo and Kim 2013).

*M. praecox* has been studied on screening of freshwater fish species for their susceptibility to a betanodavirus (Furusawa et al. 2007). Fertilized eggs of this species had oil droplets and fixing filaments for adhesion and embryoic development was similar to that of other species in the genus *Melanotaenia* (Radael et al. 2014). But there is no research on the ultrastructure of fertilized egg envelope because it is hard to get fertilized eggs from this species in aquarium. Therefore, we studied the morphology of fertilized egg with adhesive filaments, and the ultrastructures of micropyle, outer surface, inner surface and section of fertilized egg envelopes under the light and electron microscopes to find out species specificity in dwarf rainbowfish, *M. praecox* belong to family Melanotaeniidae.

## Materials and methods

### Animals

Three pairs of dwarf rainbowfish, *Melanotaenia praecox* (total length: 4–6 cm) used in this study were purchased from TrofishNet Aquarium (Yongin, Korea). The tap water used for rearing was treated with carbon filter (Pre-carbon filter, filter114 Co. Ltd., Korea) to remove chlorine, and its temperature and pH were maintained at 25 ± 0.5 °C and 6.5 ± 0.5, respectively. Biological filtration was performed using a sponge filter (Tetra TwinBrillant Super Filter™, Tetra Co. Ltd., Germany), and scraps and excrement settled on the bottom of the water tank were eliminated by exchanging one-third of the water every day. The artificial light was illuminated for 10 h per day to simulate a daytime environment using an electronic timer, and hatched *Artemia* nauplii (Great Salt Lake *Artemia* cysts, Saunders, U.S.A.) were provided as food three times a day at 9 a.m., 1 p.m., and 5 p.m.

### Collection of fertilized eggs

It used the same rearing water as water of breeding tank (45X30X30 cm) and a bundle of acrylic knitting yarn was used as a spawning ground. The fertilized eggs were isolated using fingers being careful not to break the fertilized eggs from a bundle of acrylic knitting yarn. Fertilized eggs that confirmed the formation of perivitelline space were measured for size (*n* = 30) under digital microscope (AD-7013MZT, Dino-Lite, Anmo, Taiwan) and used in this study as experimental samples.

### Electron microscopy

For scanning electron microscope (SEM) observation, first fertilized egg envelopes were pierced a hole with 1 mL injection needle and fixed in 2.5% glutaraldehyde in 0.1 M phosphate buffer (pH 7.4) for 24 h at 4 °C. After prefixation, the specimens were washed twice in the same buffer solution for 20 min. and then postfixed in 1% osmium tetroxide solution in same phosphate buffer solution for 2 h at room temperature. Specimens were dehydrated in ethanol and the samples were replaced with tert-butyl alcohol and dried with freeze dryer (ES-2030, Hitachi, Japan). The samples were coated with Pt by ion sputter (E-1045, Hitachi, Japan). Subsequently, the fertilized eggs were observed under the table top scanning electron microscope (TM-1000, Hitachi, Japan).

For transmission electron microscope (TEM) observation, prefixation, postfixation and dehydration were conducted by following the same procedure as that for SEM., cleared in propylene oxide, and embedded in an Epon 812 mixture. Ultrathin sections of embedded fertilized egg envelope were taken with an ultramicrotome (Ultracut E, Reichert-Jung, Austria) at a thickness of about 60 nm. The ultrathin sections were mounted onto copper grids, double stained with uranyl acetate followed by lead citrate, and observed with a transmission electron microscope (JEM-1400, JEOL, Japan).

## Results and discussion

### Structure of fertilized eggs

**Fig. 1:**
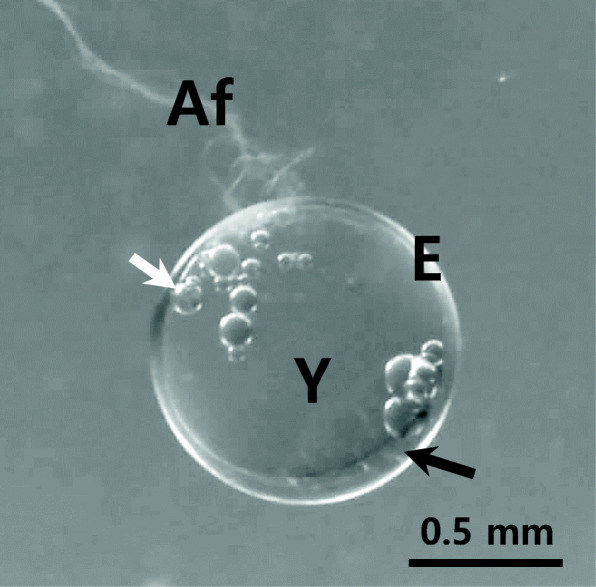
Fertilized egg of dwarf rainbowfish (*Melanotaenia praecox*). White arrow; lipid droplets, E; egg envelope, Y; yolk. The perivitelline space (black arrow) was very small and a bundle of adhesive filament (Af) was only at the area of animal pole

The fertilized eggs of *Melanotaenia praecox* were spherical with adhesive filament, transparent, demersal, and had a small perivitelline space and small oil droplets (Fig. 1). The size of fertilized egg was 1.02 ± 0.18 mm (*n* = 30). The fertilized eggs were hard enough to handle it with fingers. Fertilized egg itself had no adhesive property, but the filaments continuously have been maintained the adhesive property after sample preparation for scanning electron microscopic observation too. The fertilized eggs of most species belong to Belontiidae, Characidae, Cyprinidae and Cichlidae have adhesive property, but fertilized eggs of *Hemigrammus caudovittatus* (Characidae) and *Danio rerio* (Cyprinidae) are non-adhesive type (Kim et al. 1996; Joo and Kim 2013).

The fertilized eggs have same morphology according to the genus or family (Deung et al. 1997; Kim et al. 1999; Joo and Kim 2013; Kwon et al. 2017; Choi et al. 2019). The perivitelline space of fertilized egg of *M. praecox* was very small enough to vitelline membrane of egg stick to the fertilized egg envelope. The size of perivitelline space was related with their spawning habits. Most egg scatter species such as Cyprinidae and Characidae have a large perivitelline space for protection from the external physical impacts (Kim et al. 1996; Chang et al. 2019). But that of fishes belong to Belontiidae, Cichlidae, Callichthyidae, Loricariidae and Nothobranchiidae have a habit that laying eggs on a spawning ground have a small perivitelline space (Deung et al. 1997, 2000; Kim et al. 2009; Kwon et al. 2015, 2017; Choi et al. 2019; Kim 2020). The adhesiveness of fertilized egg from *Ancistrus cirrhosis* was known to disappeared after spawning excepting some parts that fertilized egg contact with other egg or spawning place (Kim 2020).

Although *Amphiprion frenatus* (Pomacentridae) and *Odontobutis obscura* (Eleotrididae) belong to different family, the fertilized eggs have a bundle of adhesive filaments similar to that of *M. praecox* (Kim et al. 1998, 2002). But the external shape is different. Fertilized eggs of *A. frenatus* and *O. obscura* are long ellipsoidal but that of *M. praecox* is spherical. Also, small oil droplets were in vitelline membrane, these small oil droplets are thought to be used as a nutrient to embryonic development. In species belong to Belontiidae, the fertilized eggs have a large oil droplet that provide buoyancy for their floating (Kim et al., 1999).

As mentioned above, morphological characteristics of fertilized egg from *M. praecox* including external egg shape, a bundle of adhesive filament at the area of animal pole, small perivitelline space and oil droplets, and demersal types were showed species specificity.

### Micropyle and adhesive structures

**Fig. 2:**
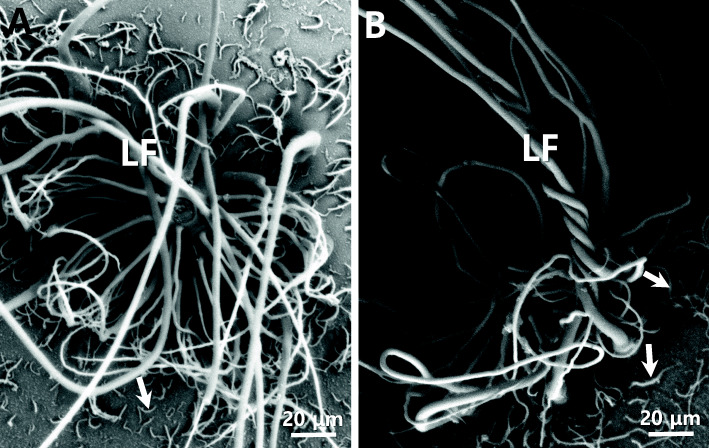
Long adhesive filaments (LF) from the fertilized eggs of *M. praecox* were only at the area of the animal pole including a micropyle (**a**) and the short adhesive filaments (arrows) were around long adhesive filaments. Sometimes the long adhesive filaments were coiled together (**b**)

Adhesive filaments were only near the micropyle on the animal pole of fertilized egg (Fig. 2a). Sometimes the long adhesive filaments were coiled together (Fig. 2b). There were two kinds of adhesive filaments, one was long whippy adhesive filaments (diameter in the middle 12.22 ± 0.52 μm, *n* = 20) and the other was short adhesive filaments (diameter in the middle 1.99 ± 0.23 μm, *n* = 20).

**Fig. 3:**
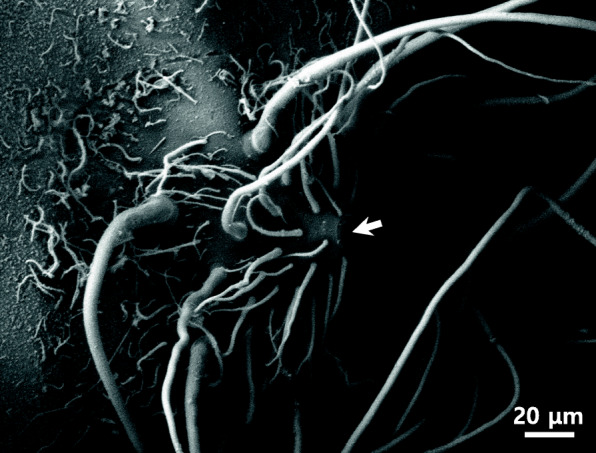
Scanning electron micrograph of micropyle (arrow) on the fertilized egg envelopes from *M. praecox*. The micropyle was conical shaped with whippy adhesive filaments

But there were no adhesive filaments in other part excepting the animal pole area. In teleost, there are adhesive fertilized eggs without adhesive filaments (Kwon et al. 2017; Choi et al. 2019) and with adhesive long filaments. (Kim et al. 1998, 2002; Kwon et al. 2017) or adhesive reticular fibers (Deung et al. 2000, Kim et al. 2009). *Nothobranchius foerschi* and *Nothobranchius rachovii* belong to Nothobranchiidae have a lot of adhesive whip-like structures over the whole outer surface of fertilized egg in both species (Kwon et al. 2017). The fertilized eggs of tomato clown anemonefish and dark sleeper have a bundle of adhesive filament. But the researchers were unable to find a micropyle using a stereo microscope and a scanning electron microscope (Kim et al. 1998, 2002). In our research, a micropyle was found in a bundle of adhesive filaments (Fig. 3). The micropyle was conical shaped with whippy adhesive filaments. The external diameter of micropyle was about 12.5 μm, and inner diameter was about 4.73 μm. In most egg with a bundle of adhesive filaments, the micropyle was believed to be located in a bundle of adhesive filaments. In some species, these adhesive filaments plays a role of protection of water loss when the water level is lowered by the tide (Dumont and Brummet, 1980). Even if it’s the same species, the morphology was different from distribution (Brummett and Dumont 1981).

Micropyle location in the area of animal pole is suitable for fertilization because the animal pole contains cytoplasm with nucleus. In general, there are no any special structures around the micropyle, but fertilized eggs of some species have special structures in the vicinity of a micropyle. In Characidae, the micropyle have a spoke-like structure consisted of protuberance lines of egg envelope in all species (Kim et al. 1996, 2005; Chang et al. 2019). The micropyle of pale chub belong to Cyprinidae is surrounded by five peaks of hill structures (Deung et al. 2000). The micropyles were same funnel shape in Belontiidae (Kim et al. 1999) and a plate coral mouth shape in genus *Nothobranchius* (Kwon et al. 2017). Therefore, the structure of micropyle seems to be family, genus or species specificities.

### Outer surface of the fertilized egg envelopes

**Fig. 4:**
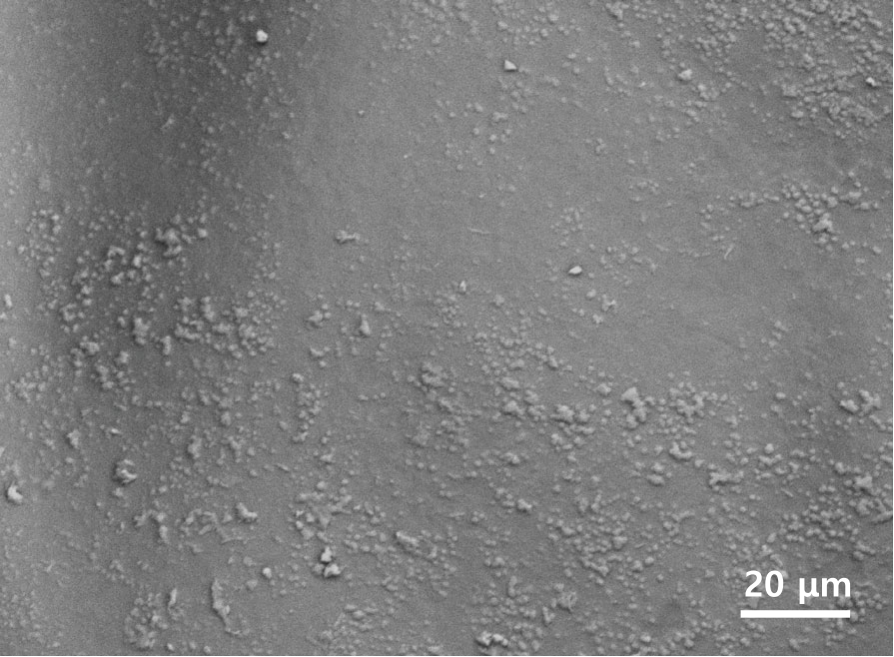
The outer surface of fertilized egg envelope. The outer surface was rough and there were no adhesive filaments on other side of egg envelope excepting micropylar region

In this study, the outer surface was rough side without adhesive filaments excepting micropylar region (Fig. 4). The outer surface of fertilized egg was smooth and the egg envelope have a bundle of adhesive filaments (Kim et al. 1998, 2002). This difference of outer surface could be species specificity. The ultrastuctures of outer surface of fertilized egg envelope were vary according to the species, genus and family.

In the fishes belong to Callichthyidae, Cichlidae and Belontiidae, the ultrastructure of outer surface of egg envelope was showed family specificity because the structure of outer surface was same according to the family (Deung et al. 1997; Kim et al. 1999; Choi et al. 2019). That of Callichthyidae have adhesive protuberances (Choi et al. 2019), that of Cichlidae have covered with adhesive reticular structures (Deung et al. 1997; Kim et al. 2009), and that of Belontiidae have many grooves (Kim et al. 1999). But the fine structure of outer surface of fertilized egg envelope were different according to the species in Cyprinidae. That of *Tanichthys alborubes* have rod-like structures (Kim et al. 1998), that of pale chub have Indian club-like structures (Deung et al. 2000), that of *Hemibarbus longirostris* have taste bud-like structures (Kim et al. 2001), and that of *Danio rerio* have knob-like structures (Joo and Kim 2013). Therefore, ultrastructures of outer surface of fertilized egg envelope was showed genus specificity in fishes belong to Cyprinidae. The ultrastructures of outer surface can be different in same family or same in different family (Kim et al. 1996, 2005). Also the structure of outer surface of egg envelope could be changeable by species variation (Joo and Kim 2013).

### Fine structure section of fertilized egg envelope

**Fig. 5:**
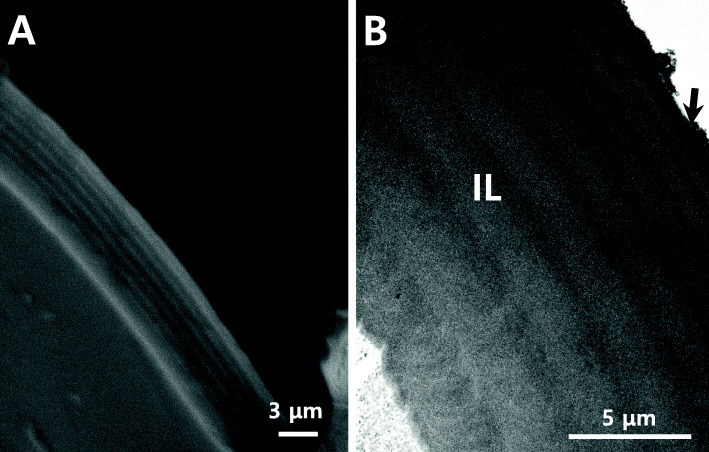
The electron micrographs of the fertilized egg envelopes section. The fertilized egg envelope was a multi-layer lamellar structure in SEM image (**a**). The egg envelope consisted of two layers, an inner lamella layer (IL) and an outer layer with high electron-density (arrow). And the inner layer was 8 layers on TEM image (**b**)

Under the scanning electron microscope, the section of fertilized egg envelope from *M. praecox* was a multi-layer and the thickness was about 74.46 ± 0.41 μm (*n* = 20) (Fig. 5a). Also, the fertilized egg envelopes consisted of 2 layers, an inner lamellae layer and an outer layer with high electron-density on TEM image. And the inner layer was 8 layers (Fig. 5b). The inner lamellar layer tended to be wide on the outside and narrow on the inside in particular. But this inner lamellar layer had equal spacing in all species belong to Cichlidae (Kim et al. 1999).

In general, the number of layer of fertilized egg envelope was different according to the family or species but sometimes it is same in a family such as Belontiidae, Cichlidae, Callichthyidae, and Nothobranchiidae (Kim et al. 1999, 2009; Kwon et al. 2015, 2017; Choi et al. 2019). The fertilized egg envelope consisted of 2 or 3 layers in most teleost and the inner layer was showed lamellar structure alternated high and low layers due to the difference of the electron-density. Unusually, the fertilized egg envelope from *Ancistrus cirrhosis* (Loricariidae) have counter structure from other species (Kim 2020). In species belong to Characidae and Cyprinidae, the fertilized egg envelopes have different structure according to the species. In Characidae, those of head and tail light fish, serape tetra, Buenos aires tetra consisted of 3 layers, and those of black tetra and glowlight tetra consisted of 2 layers. But the number of inner layer is different according to the species. The inner layers of head and tail light fish and glowlight tetra consisted of 3 layers, that of black tetra consisted of 4 layers, that of Buenos aires tetra consisted of 5 layers, and that of serape tetra consisted of 5–6 layers (Kim et al. 1996, 2005; Chang et al. 2019). As mentioned above, the number of layers on fertilized egg envelope or section structure are showed species specificity, genus specificity or family specificity. But more structural research on the other species belong to Melanotaeniidae is needed to determine whether it is family specificity or not.

The structure of egg envelope was related with external environment including intensity of radiation, hydraulic pressure, water wave and current, and spawning behavior. The thickness of egg envelope was known to be thick in floating type than demersal (Stehr and Hawkes 1979), in oviparity than ovoviparity (Riehl and Greven 1993) and in the fast stream (Ivankov and Kurdyayeva 1973).

## Conclusions

The fertilized eggs of dwarf rainbowfish (*Melanotaenia praecox*) belong to Melanotaeniidae were spherical with adhesive filament, transparent, demersal, and had a small perivitelline space and oil droplets. And there were two kinds of adhesive filament, those of long and thick, and short and thin on the fertilized eggs. A micropyle was conical shaped with adhesive filament and located at the area of the animal pole. The outer surface of fertilized egg was rough side. Also, the fertilized egg envelope consisted of two layers, an inner lamellae layer and an outer layer with high electron-density. And the inner layer was 8 layers. Collectively, these morphology of fertilized egg including egg size, adhesive property, oil droplets, small perivitelline space, a bundle of adhesive filaments on the animal pole, and ultrastructural characteristics of egg envelope including micropyle, outer surface, thickness of envelope, and the number of layers were showed species specificity.

## Data Availability

No applicable.
